# Investigating the utility of COVID-19 antibody testing in end-stage renal disease patients receiving haemodialysis: a cohort study in the United Kingdom

**DOI:** 10.1186/s12882-021-02366-2

**Published:** 2021-04-27

**Authors:** Olivia Wickens, Rajkumar Chinnadurai, Fahmida Mannan, Frida Svendsen, Mirza Yasar Baig, Chukwuma Chukwu, Ibrahim Ali, Christina Summersgill, Dawn Evans, Berckley V. Antoine, Julie Oxton, Nathan Mairs, Emma Flanagan, Robert Oliver, Philip A. Kalra, Dimitrios Poulikakos

**Affiliations:** 1 Department of Renal Medicine, Salford Royal NHS Foundation Trust, Salford, M6 8HD UK; 2 Faculty of Biology, Medicine and Health, University of Manchester, Manchester, UK; 3 Research and Innovation, Salford Royal NHS Foundation Trust, Salford, UK

**Keywords:** COVID-19, Antibody testing, Haemodialysis, End-stage renal disease

## Abstract

**Background:**

End-stage renal disease (ESRD) patients receiving haemodialysis (HD) are a vulnerable group of patients with increased mortality from COVID-19. Despite improved understanding, the duration of host immunity following COVID-19 infection and role of serological testing alone or in addition to real-time reverse transcription polymerase chain reaction (rRT-PCR) testing in the HD population is not fully understood, which this study aimed to investigate.

**Methods:**

There were two parts to this study. Between 15th March 2020 to 15th July 2020, patients receiving HD who tested positive on rRT-PCR for SARS-CoV-2 were recruited into the COVID-19 arm, whilst asymptomatic patients without a previous diagnosis of COVID-19 were recruited to the epidemiological arm of the Salford Kidney Study (SKS). All patients underwent monthly testing for anti-SARS-CoV-2 antibodies as per routine clinical practice since August 2020. The aims were twofold: firstly, to determine seroprevalence and COVID-19 exposure in the epidemiological arm; secondly, to assess duration of the antibody response in the COVID-19 arm. Baseline characteristics were reviewed between groups. Statistical analysis was performed using SPSS. Mann-Whitney U and Chi-squared tests were used for testing significance of difference between groups.

**Results:**

In our total HD population of 411 patients, 32 were PCR-positive for COVID-19. Of the remaining patients, 237 were recruited into the SKS study, of whom 12 (5.1%) had detectable anti-SARS-CoV-2 antibodies. Of the 32 PCR-positive patients, 27 (84.4%) were symptomatic and 25 patients admitted to hospital due to their symptoms. Of the 22 patients in COVID-19 arm that underwent testing for anti-SARS-CoV-2 IgG antibodies beyond 7 months, all had detectable antibodies.

A higher proportion of the patients with COVID-19 were frail compared to patients without a diagnosis of COVID-19 (64.3% vs 34.1%, *p* = 0.003). Other characteristics were similar between the groups. Over a median follow up of 7 months, a higher number of deaths were recorded in patients with a diagnosis of COVID-19 compared to those without (18.7% vs 5.9%, *p* = 0.003).

**Conclusions:**

Serological testing in the HD population is a valuable tool to determine seroprevalence, monitor exposure, and guide improvements for infection prevention and control (IPC) measures to help prevent local outbreaks. This study revealed HD patients mount a humoral response detectable until at least 7 months after COVID-19 infection and provides hope of similar protection with the vaccines recently approved.

**Supplementary Information:**

The online version contains supplementary material available at 10.1186/s12882-021-02366-2.

## Background

Since the discovery of Coronavirus disease 2019 (COVID-19) in December 2019, caused by severe acute respiratory syndrome coronavirus-2 virus (SARS-CoV-2), we have witnessed a global pandemic. SARS-CoV-2 has become the seventh coronavirus to infect humans and the third identified coronavirus to cause a major outbreak in humans [1].

End-stage renal disease (ESRD) patients receiving haemodialysis (HD) have been identified as a particularly high-risk group of patients at increased risk of mortality from COVID-19 [2–4]. This is because many dialysis patients have underlying chronic co-morbidities, are often of an older age group and have an impaired immune response. In addition, maintenance HD patients have significantly increased risk of exposure to SARS-CoV-2 as they are unable to self-isolate, having to attend frequent HD sessions, usually thrice per week, with associated risks during transport.

Molecular testing via respiratory tract swabs, analysed by real-time reverse transcription polymerase chain reaction (rRT-PCR), remains the gold-standard diagnostic test for suspected COVID-19. However, false-negatives can occur with an insufficient sample quantity of viral genome, improper sampling or missing the window period of viral replication [5]. More recent tests involve viral antigen detection usually from nasopharyngeal (NP) swabs, which can provide results within 15 min [6]. Both viral nucleic acid and viral antigen tests only test for the presence of active infection and have no role in the identification of past infection, although they have been reported to continue to remain positive due to prolonged viral shedding for up to 63 days following the onset of symptoms [7, 8]. With high numbers of asymptomatic and pre-symptomatic cases or viral shedding post-infection, keeping infection transmission under control continues to remain an enormous challenge.

With the limitations of molecular testing for COVID-19, there has been much interest in the use of serological anti-SARS-CoV-2 antibody testing. With a broad spectrum of clinical presentation of COVID-19 from asymptomatic infection, or mild flu-like symptoms through to acute respiratory distress syndrome (ARDS), multi-organ failure and death, serological testing plays an important role in surveillance, epidemiological studies and as an indirect marker of infection. Serological testing can be used to monitor disease prevalence and evaluate screening measures and protocols aiming at limiting transmission within dialysis units. Additionally, serological testing is also essential to quantify the level and duration of antibody response after COVID-19 infection, as with loss of detectable anti-SARS-CoV-2 antibodies and short-lived humoral immunity there may be a risk of potential reinfection or viral reactivation, particularly whilst patients are awaiting vaccination [9]. This is of particular concern in the vulnerable category of ESRD patients receiving maintenance HD, which are a high-risk group of patients at increased morbidity and mortality from infection with SARS-CoV-2 due to their impaired immune responses to infection and vaccination [10].

To this end, this study aimed at determining the IgG seroprevalence among both asymptomatic patients without a diagnosis of COVID-19 and symptomatic or asymptomatic rRT-PCR positive patients in our HD population. Additionally, serological testing was used to assess the duration of antibody response and immunity in those infected with COVID-19. Baseline characteristics were compared between patients who tested positive and those who tested negative for SARS-CoV-2 by rRT-PCR to determine if there were particular risk factors for infection.

## Methods

Between 15th March 2020 to 15th July 2020, patients receiving HD who tested positive on rRT-PCR for SARS-CoV-2 were recruited into the COVID-19 arm, whilst the remainder of patients were recruited to the epidemiological arm of Salford Kidney Study (SKS). SKS is a prospective observational study in the United Kingdom which has recruited chronic kidney disease patients since the year 2002. The ethical approval of SKS has been extended to include dialysis patients (both HD and peritoneal dialysis (PD)) since 2016. This research work has been performed in accordance with the Declaration of Helsinki and SKS has ethical approval obtained from the North West - Greater Manchester South Research Ethics Committee, UK (reference number: 15/NW/0818). All 269 patients involved in our observational analysis have signed an informed consent.

Details of patients recruited into SKS are elaborated in the Research Registry (https://www.researchregistry.com; researchregistry5962). In brief, this is longitudinal epidemiological study that involves annual blood sampling with samples processed and stored at − 75 °C for subsequent research analysis (EDTA whole blood, serum, plasma, and citrate plasma). All adult patients who have provided informed consent are recruited to the SKS.

The protocol was amended to include a sub-study for COVID-19 positive patients in order to investigate the time course of development of antibodies and the longevity of antibody response in HD patients. This sub-study included collection and storage of blood samples at recruitment (at or shortly after COVID-19 infection) and at intervals of 8 to 14 days on five occasions after infection, followed by six monthly interval samples. From August 2020 onwards, maintenance HD patients underwent COVID-19 IgG antibody testing at monthly intervals as per newly implemented routine clinical practice.

### Serological testing

All sera collected was tested only for COVID-19 IgG antibody. The initial antibody tests were obtained on recruitment and the criterion for recruitment was being asymptomatic and not having a previous diagnosis of COVID-19. This initial research assay was measured via a CE marked chemiluminescent immunoassay (SNIBE, Shenzhen, China) and the analysis was performed by Medical Diagnostics Ltd. in conjunction with Affinity Biomarker Labs, with a result of > 1 AU/ml deemed positive [11]. The calculated clinical sensitivity and specificity according to manufacturer for the chemiluminescent analytical assay for SARS-CoV-2 IgG antibody was 91.21, and 97.3%, respectively [11]. Subsequent antibody tests were performed via the Public Health England (PHE) approved Siemens’ immunoassay using acridinium ester chemiluminescent technology [12], which became available in the hospital laboratory from 12th June 2020. From August 2020, monthly screening surveillance with COVID-19 IgG antibody testing with Siemens’ immunoassay of the total HD cohort was implemented. An index ≥1.0 was deemed positive for the Siemens’ assay based on the manufacturer’s assigned cut-off and sensitivity and specificity for the Siemens’ assay were reported at 98.1 and 99.9% respectively [12].

### rRT-PCR testing

Initial rRT-PCR testing was performed if a patient was suspected to have COVID-19 or was contact case of a person with confirmed COVID-19. A suspected case was defined as a person exhibiting symptoms and signs based on PHE criteria on screening prior to HD (or self-presentation). A contact case was defined as a person who received HD during the same or subsequent shift (possible contact in waiting area) if there were greater than two positive cases in one shift. Suspected cases would be transferred directly to the main base dialysis unit (Salford Royal Hospital) for testing and assessment and for their next dialysis sessions. If the initial rRT-PCR was negative for SARS-CoV-2 it was repeated at their following HD session, and if negative again and asymptomatic, they would return to their satellite unit. COVID-19 identification was performed via an upper respiratory tract swab for SARS-CoV2 by rRT-PCR. Screening with nasopharyngeal swabs by rRT-PCR for asymptomatic contact cases was performed in the satellite HD units. rRT-PCR remains the gold standard test for COVID-19, however its sensitivity depends on the patterns of viral shedding and the probability of a positive result varies over the course of infection. There is heterogeneity in reported accuracy from different studies with reported false negative rate ranging from 13% in symptomatic patients [13], to 30–40% in close contacts of confirmed cases against serological diagnosis with SARS-CoV-2 antibodies [14]. False-positive rates have been reported between 0.8 and 4.0% [15].

### Data collection

Data was collected at study baseline (rRT-PCR date between March and July 2020) from the electronic patient records. This included demographics, body mass index (BMI), clinical frailty score (CFS) and comorbidities, including a history of diabetes and cardiovascular events. Blood results including full blood count, ferritin, and albumin were recorded at baseline or within 2 weeks from the monthly dialysis bloods for all available patients. All patients were followed up from study baseline to endpoints which included death, loss to follow up or an arbitrary study end date of 31st December 2020.

### Study definitions

The comorbidity, medication and frailty data were collected at the time of the first antibody test date. A smoking history was defined as a patient reported history of smoking, irrespective of the number of cigarettes smoked. Cardiovascular disease (CVD) history included a composite of non-fatal cardiac arrest, acute coronary syndrome, myocardial infarction, peripheral vascular disease, cerebrovascular accident and congestive cardiac failure. Renin-angiotensin system inhibitors (RASi) medications included angiotensin converting enzyme inhibitors and angiotensin receptor blockers. Frailty status was determined using the CFS, with any patient with a score of five and above on the CFS was defined as being frail [16, 17].

### Statistics

Statistical analysis was performed using SPSS version-23, licenced to the University of Manchester. Throughout the analysis, categorical values were expressed as number (%), and the *p*-value was derived using the Chi-square test. As most of the continuous values were non-normally distributed, they were expressed as median (interquartile range) and the Mann-Whitney U test was used to calculate the *p*-value. Cox-regression analysis was used to study the strength of association between COVID-19 rRT-PCR positivity and all-cause mortality. To overcome the influence of competing risks, hazard ratios were derived by censoring at the competing event. (i.e., negative patients developing COVID-19 infection during follow-up). A p-value < 0.05 was considered statistically significant in this study [18].

## Results

### Seroprevalence of COVID-19 in asymptomatic rRT-PCR-negative HD population

**Fig. 1:**
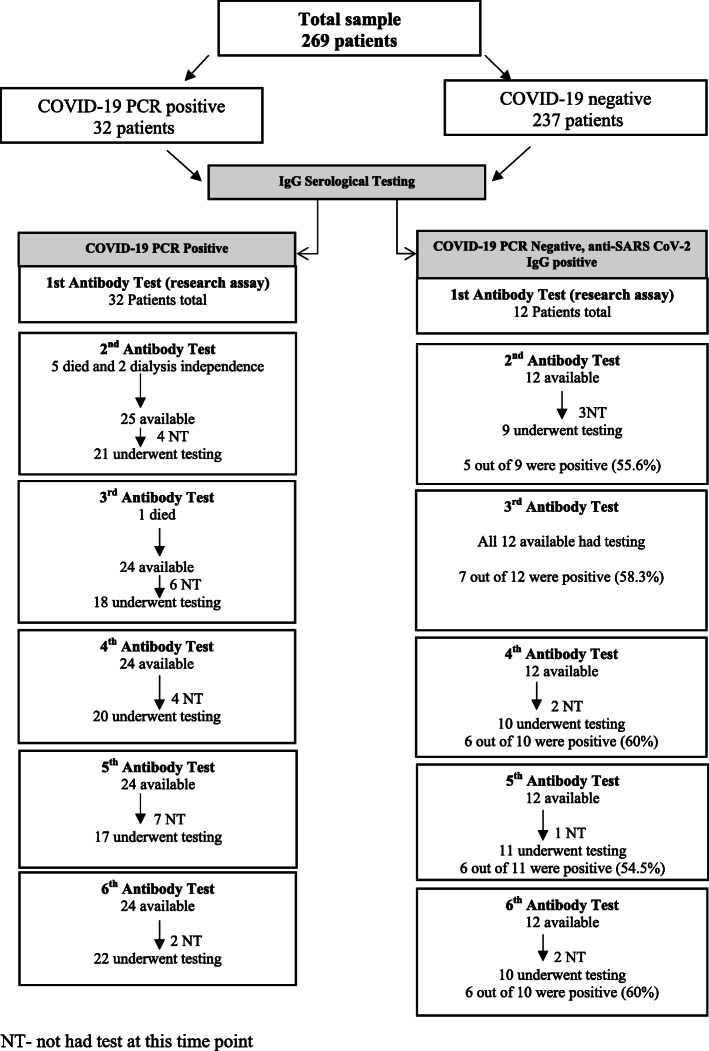
Representation of serological testing at various time points in SARS-CoV-2 seropositive patients

A total of 237 asymptomatic HD patients without a diagnosis of COVID-19 consented out of a total HD population of 411. All 237 patients were recruited to the epidemiological arm of SKS. In this group of patients blood samples obtained for the first antibody testing at recruitment were analysed via the chemiluminescent immunoassay performed at a median of 3 days (interquartile range (IQR) -2 to 14) from the rRT-PCR test. The seroprevalence of IgG to SARS-CoV-2 was 5.1% (*n* = 12) in this group (Table 1). All 237 patients also had a negative rRT-PCR test between April and July 2020. The second antibody testing performed via the Siemens’ antibody assay was at a median of 62 days (IQR 47–77) from the negative rRT-PCR test result. Of the 12 rRT-PCR negative patients who had detectable anti-SARS-CoV-2 antibodies on the first antibody test, antibodies were detectable in 54.5–60% of patients at the monthly serological testing points during the follow-up period with the Siemens’ assay (Fig. 1). Ten patients tested positive for COVID-19 on rRT-PCR testing during the follow-up period.

**Table 1:** Antibody status and COVID-19 rRT-PCR positivity correlation at baseline (first antibody)

Variable	Total 269	COVID-19 PCR positive (32)	COVID-19 PCR negative (237)
Number of patients with IgG antibody positive	43 (16%)	31 (96.9%)	12 (5.1%)
Time between Covid-19 PCR and 1st antibody level, days	4 (1–17)	22 (16–36)	3 (−2 to 14)

Categorical variables are expressed as number (%). Continuous variables are expressed as median (interquartile range)

*rRT-PCR* real-time reverse transcription polymerase chain reaction

### Serological testing in COVID-19 rRT-PCR-positive patients

A total of 32 patients receiving HD who tested positive for SARS-CoV-2 by rRT-PCR were recruited into COVID-19 arm of the SKS. Twenty-seven patients (84.4%) were symptomatic for COVID-19 and 25 patients (78.1%) had a COVID-19 associated hospital admission. A COVID-19 associated hospital admission included patients who either tested positive on the date of hospital admission or during their admission. The first baseline antibodies were tested via the CE marked chemiluminescent immunoassay (SNIBE, Shenzhen, China) and the analysis was performed by Medical Diagnostics Ltd. in conjunction with Affinity Biomarker Labs. Of the 32 patients who had antibody testing at baseline, 31 (96.9%) had detectable IgG to SARS-CoV-2. This was performed at a median of 22 days (IQR 16–36) from the positive rRT-PCR test result. Subsequent antibody testing from the second antibody testing point onward was performed via the Siemens’ assay. Sera for COVID-19 antibodies were collected at regular time points, up to 6 times (median number of days prior to obtaining second, third, fourth, fifth and sixth samples were 120, 152, 185, 215 and 242 respectively), as seen in Table 2.

**Table 2:** Trend of antibody status in the 32 positive patients

Variable	First antibody	Second antibody	Third antibody	Fourth antibody	Fifth antibody	Sixth Antibody
Number of patients with IgG antibody level available	32	21	18	20	17	22
Number of patients with IgG antibody level positive	31 (96.9%)	19 (90.5%)	17 (94.4%)	18 (90%)	15 (88.2%)	22 (100%)
Median time between Covid-19 PCR and antibody, days	22 (16–36)	120 (109–124)	152 (142–158)	185 (174–189)	215 (210–220)	242 (233–250)

Categorical variables are expressed as number (%). Continuous variables are expressed as median (interquartile range). The first antibody testing was performed via a CE marked chemiluminescent immunoassay (SNIBE, Shenzhen, China) and the analysis was performed by Medical Diagnostics Ltd. in conjunction with Affinity Biomarker Labs. All subsequent antibody testing was performed by Siemens’ immunoassay using acridinium ester chemiluminescent technology which was a Public Health England approved hospital assay)

Of the 32 patients, one patient had undetectable antibodies from the second antibody test onwards. Another patient had undetectable antibodies from the second antibody until the sixth antibody test in which antibodies were then detected, which is the reason for the reduced percentage of patients with detectable antibodies at some of the sampling time points. In the sixth antibody collection point all the patients who underwent testing at this timepoint (*n* = 22) had detectable antibodies, with the one patient who had undetectable antibodies from the second test onwards not having testing at this period. There was also one patient who had undetectable antibodies on the first antibody test, who then was found to have positive antibodies on all subsequent testing.

After the first antibody testing, there was a decline in the number of COVID-19 rRT-PCR-positive patients who underwent serological testing during the follow-up period (*n* = 21; *n* = 18; *n* = 20, *n* = 17, *n* = 22 at the second, third, fourth, fifth and sixth antibody samples respectively). The decline in patient numbers were due to death (*n* = 6), dialysis independence (*n* = 2) and not all samples were retrieved at the scheduled time points due to practical challenges related to the pandemic (transfer of patients between units and impact of the pandemic on research capacity), (Fig. 1).

Of the 22 rRT-PCR-positive patients that were revealed to have detectable antibodies at a median of 242 days (IQR 233–250) from PCR positivity, 17 were symptomatic with COVID-19, and 16 had a COVID-19 associated hospital admission. Although the number of patients with detectable IgG antibodies to SARS-CoV2 varied over the course of the study, seropositivity remained at least 88.2% or above at each sampling point. There were a total of 6 deaths during the follow-up period, of which 2 were secondary to COVID-19 infection. While not statistically significant, there was a trend towards a higher risk of all-cause mortality among patients who initially tested positive for COVID-19 via rRT-PCR at baseline after adjustment for possible confounders (HR 2.29, 95% CI: 0.72–7.35). Therefore, COVID-19 infection was not an independent risk factor associated with all-cause mortality during the study period of interest (Supplementary Table 1).

### Comparison of baseline characteristics of COVID-19 rRT-PCR-negative and rRT-PCR-positive patients

Baseline characteristics for the study population are presented as a comparison between these two groups in Table 3. The median age of the study population was 61 years (IQR 50–73) and a predominance of males and Caucasian ethnicity, though not significantly different between the groups. The groups were similar in the baseline clinical characteristics, including smoking history, body mass index (BMI), history of diabetes and cardiovascular diseases, apart from the proportion of patients in the COVID-19 positive group were more clinically frail (64.3% vs 34.1%; *p* = 0.003). Over a median follow up of 7.6 months, a higher proportion of deaths was observed in the COVID-19 positive group (18.7% vs 5.9%; *p* < 0.003), although 10 patients in the rRT-PCR negative group tested positive for COVID-19 on rRT-PCR in the follow-up period, of which three required hospital admission.

**Table 3:** Baseline demographics of the recruited haemodialysis patients

Variable	Total patients (269)	COVID-19 rRT-PCR positive (32)	COVID-19 rRT-PCR negative (237)	***p***-value positive vs negative
Age, years	61 (50–73)	62 (54–75)	60 (50–73)	0.663
Gender, male	177 (65.7%)	22 (68.7%)	150 (63.3%)	0.546
Ethnicity, Caucasian	196 (72.8%)	25 (78%)	171 (72.2%)	0.476
BAME	73 (27.2%)	7 (22%)	66 (27.8%)	
Smoking history	32 (11.9%)	8 (25%)	24 (10.1%)	0.344
Weight, Kg	76 (64–89.5)	81.5 (63–95)	75 (65–89)	0.405
BMI, Kg/m^2^	27 (23–31)	26.5 (22.5–32)	27 (24–31)	0.939
Diabetes mellitus	97 (36.1%)	9 (28.1%)	88 (37.1%)	0.319
CVD	60 (22.3%)	8 (25%)	52 (21.9%)	0.696
RASi	93 (34.6%)	9 (28.1%)	84 (35.4%)	0.414
Frail (CFS>/=5), *n* = 189	73/189 (38.6%)	18/28 (64.3%)	55/161 (34.1%)	**0.003**
Dialysis vintage, months	26 (11–64)	42 (14–71)	25 (10–61)	0.658
Dialysis access, AVF	167 (62.1%)	21 (65.5%)	146 (61.6%)	0.701
URR, %	72 (65–78)	69 (66–77)	73 (67–78)	0.162
Albumin, g/L, *n* = 262	37 (34–40)	38 (34–40)	37 (34–40)	0.873
Ferritin, ng/mL, *n* = 262	367 (208–652)	364 (239–706)	368 (208–647)	0.761
TSats, %, *n* = 262	24 (16–34)	27 (17–36)	24 (16–34)	0.418
Vitamin D level, nmol/L, *n* = 211	43 (28–55)	42 (24–53)	37 (30–71)	0.613
Haemoglobin, g/L, *n* = 214	107 (96–117)	103 (86–120)	107 (96–117)	0.616
WCC, ×10^9^/L, *n* = 214	7 (5–8)	6.6 (4–9)	7 (5–8)	0.925
Lymphocytes, ×10^9^/L, *n* = 214	1.1 (0.7–1.45)	0.75 (0.5–1.2)	1.10 (0.8–1.5)	0.005
Platelet count, ×10^9^/L, *n* = 214	204 (157–256)	189 (117–262)	207 (158–254)	0.286
**Outcomes on follow-up**				
Follow up, months	7.6 (6.9–8.1)	8.7 (8.1–8.9)	7.2 (6.8–8)	**< 0.001**
COVID-19 associated hospital admission	28 (10.4%)	25 (78.1%)	3 (1.26%)	**< 0.001**
ICU admission	3	3	0	–
Deaths	18 (6.7%)	6 (18.7%)	12 (5.9%)	**0.003**

Categorical variables are expressed as number (%), and the p-value was derived using the Chi-square test. Continuous variables are expressed as median (interquartile range) and the Mann-Whitney U test was used to calculate the *p*-value

*BAME* black Asian minority ethnicity, *BMI* body mass index, *CVD* cardiovascular disease, *RASi* renin-angiotensin system inhibitors, *CFS* clinical frailty score, *AVF* arterio-venous fistula, *URR* urea reduction ratio, *WCC* white cell count, *TStats* transferrin saturations, *ICU* intensive care unit

## Discussion

Our study has revealed a seroprevalence of 5.1% in the maintenance, asymptomatic HD population. In the 22 of the 32 COVID-19 rRT-PCR-positive patients who had antibodies tested beyond 7 months, 100% still had detectable anti-SARS-CoV-2 antibodies. The majority of baseline characteristics were similar between both COVID-19 rRT-PCR negative and positive patients, although there was a statistically higher prevalence of frailty in the rRT-PCR positive group.

Patients with ESRD receiving HD, in particular in-centre dialysis patients, have a higher chance of acquiring COVID-19 infection due to their regular contacts with health care personnel and other people when they attend for their dialysis sessions. They are a vulnerable group of patients who are at risk of severe COVID-19 disease due to their comorbidities and frailty [2, 19]. Serological testing is key in monitoring seroprevalence in this high-risk category of patients, enabling continuing review and monitoring of current infection prevention and control (IPC) measures. A previous serosurvey from 316 healthcare workers in our centre during the period of this study demonstrated 6% seroprevalence in healthcare workers directly involved in patient care [20]. Our results revealed a slightly lower seroprevalence compared to the contemporaneous seroprevalence in healthcare workers within the department and healthy adult blood donors within the North West of the UK (6.4%) [21]. The lower seroprevalence is likely explained by the introduction of enhanced IPC measures and guidance resulting in reduced local transmission and outbreaks [22–24].

Due to the recent discovery of SARS-CoV-2, knowledge and understanding of the time host antibodies remain detectable and the immunological response to infection is limited. Out of the 32 rRT-PCR positive patients, one patient had undetectable antibodies 112 days after the rRT-PCR result on the first antibody test performed via the CE marked chemiluminescent immunoassay. It was not until 132 days after the positive rRT-PCR that anti-SARS-CoV-2 antibodies were detected in this patient via the Siemens’ immunoassay. This may have been explained by the different assays used or that the initial test may have been a false negative. In all subsequent antibody tests for this patient there were detectable antibodies.

A second patient had detectable antibodies on the first and sixth antibody testing points, with undetectable antibodies in between these timepoints, of which the reason for this inconsistency is unclear. All antibody tests for this patient except for the initial test were analysed via the Siemens’ assay. Of importance, the rRT-PCR testing in between the first and sixth antibody testing for this patient were negative, and is therefore, unlikely to have been caused by reinfection.

A third patient had undetectable antibodies from the second antibody test onwards. The duration between the positive rRT-PCR test and positive antibody test for this patient was 109 days, revealing a good initial duration of host antibody response.

It is thought that the degree of natural immunity an individual develops might be associated with severity of infection, with reports of higher antibody titres to SARS-CoV-2 in patients with a more severe clinical course of infection [25, 26]. The patient that had undetectable antibodies from the second antibody test onwards was not admitted to hospital and one possible explanation may have been that they experienced a milder clinical course of infection and subsequently a lower titre and short-lived antibody response.

A total of 22 rRT-PCR positive patients were revealed to have detectable antibodies at a median of 242 days (IQR 233–250) from PCR positivity. The results are very encouraging in that 100% of these 22 patients who underwent serological testing at greater than 7 months after initial infection had anti-SARS-CoV-2 antibodies detectable. This finding indicates that ESRD patients receiving HD are able to form antibodies against COVID-19. Of importance, patients may still have cellular immunity even when antibody testing for serological immunity is undetectable.

The limitations of the study include the use of different assays used between the first baseline and subsequent antibody tests, and the decline in the number of COVID-19 positive patients who underwent antibody testing with time.

Additionally, antibody titres were unavailable to evaluate the change in levels over time. Furthermore, there may have been a possible volunteer bias for the epidemiologic part of the study with those consented having increased adherence to PHE guidance and IPC measures. Despite these limitations, this is the first study to investigate the serial COVID-19 antibody status in HD patients over a period of at least 7 months from initial infection, with the longest duration in one patient recorded up to 264 days from the initial rRT-PCR positive swab.

Serological testing is easy to obtain from HD patients when they attend dialysis without the need for additional phlebotomy. This is an extremely valuable tool for determining seroprevalence and guiding IPC to reduce local transmission rates. It can be used to identify vulnerable patients in whom anti-SARS-CoV-2 antibodies are undetectable, whether due to lost immunity or absence of previous exposure to COVID-19, and who are potentially more susceptible to infection, be it an initial, recurrence or due to viral reactivation [9].

## Conclusions

Ongoing surveillance of asymptomatic patients with the use of serological testing is essential in continuing to reduce the risk of transmission and outbreaks amongst dialysis units and ensuring safety standards are maintained. We report a lower regional seroprevalence during the period of this study probably due to effective PHE and departmental IPC measures.

Our data has revealed that the vast majority of high-risk ESRD patients receiving maintenance HD develop a good humoral response, which in surviving patients is present beyond 7 months after infection with COVID-19, providing hope of similar protection with vaccines now recently approved. Further larger and long-term studies are warranted to provide information on the duration of immune protection against COVID-19 infection and to determine if the same response can be achieved with vaccination.

## Supplementary Information

**Additional file 1: Supplementary Table 1.** Cox- regression analysis for all-cause mortality in the whole population (Multivariate model).

## Data Availability

The datasets used and analysed during the current study are available from the corresponding author on reasonable request.

## Abbreviations

BMI: Body mass index
CFS: Clinical frailty score
CVD: Cardiovascular disease
RASi: Renin-angiotensin system inhibitors
COVID-19: Coronavirus disease 2019
ESRD: End-stage renal disease
HD: Haemodialysis
IPC: Infection prevention and control
IQR: Interquartile range
NP: Nasopharyngeal
PD: Peritoneal dialysis
PHE: Public Health England
rRT-PCR: Real-time reverse transcription polymerase chain reaction
SARS-CoV-2: Severe acute respiratory syndrome coronavirus-2 virus
SKS: Salford Kidney Study
