# Supplementary material for: Investigating the utility of COVID-19 antibody testing in end-stage renal disease patients receiving haemodialysis: a cohort study in the United Kingdom
Source: BMC Nephrol. 2021 Apr 27;22:154. doi: 10.1186/s12882-021-02366-2 (PMC8075608; doi:10.1186/s12882-021-02366-2)
Supplement: Supplementary file 1 — Additional file 1: Supplementary Table 1. Cox- regression analysis for all-cause mortality in the whole population (Multivariate model). [file 12882_2021_2366_MOESM1_ESM.docx]

**Investigating the utility of COVID-19 antibody testing in end-stage renal disease patients receiving haemodialysis: a cohort study in the United Kingdom**

**Supplementary table 1. Cox- regression analysis for all-cause mortality in the whole population (Multivariate model)**

| **Variable** | **HR (95% CI)** | **p-value** |
| --- | --- | --- |
| COVID-19 rRT-PCR positive | 2.29 (0.72-7.35) | 0.16 |
| Age, years | 1.03 (0.99-1.07) | 0.12 |
| Gender, male | 0.94 (0.35-2.49) | 0.89 |
| Ethnicity, Caucasian | 4.91 (0.63-37.94) | 0.13 |
| Smoker | 1.26 (0.42-3.82) | 0.68 |
| Diabetes mellitus | 3.62 (1.31-9.99) | **0.013** |
| Cardiovascular disease | 0.32 (0.07-1.43) | 0.14 |
| Dialysis vintage, months | 1.00 (0.99-1.01) | 0.59 |

Multivariate model adjusted for COVID-19 rRT-PCR status, age, gender, ethnicity, smoking, history of diabetes mellitus, history of cardiovascular disease and dialysis vintage
